# Retraction: Testosterone protects mitochondrial function and regulates neuroglobin expression in astrocytic cells exposed to glucose deprivation

**DOI:** 10.3389/fnagi.2024.1485294

**Published:** 2024-08-28

**Authors:** Frontiers Editorial Office

The journal retracts the 27^th^ June 2016 article cited above.

Following publication, concerns were raised regarding the integrity of the images in the published figures. An investigation was conducted in accordance with Frontiers' policies, and image duplication concerns were identified in Figure 6C. The authors failed to provide a satisfactory explanation during the investigation, the data and conclusions of the article have been deemed unreliable and the article has been retracted.

The authors agreed to this retraction. Frontiers would like to thank the users on PubPeer for bringing the published article to our attention.

